# Association of monocyte-to-high density lipoprotein ratio with arterial stiffness in patients with diabetes

**DOI:** 10.1186/s12872-021-02180-6

**Published:** 2021-07-30

**Authors:** Dyah Samti Mayasari, Nahar Taufiq, Hariadi Hariawan

**Affiliations:** 1 Department of Cardiology and Vascular Medicine, Public Health and Nursing, Faculty of Medicine, Universitas Gadjah Mada, Jl Farmako No 1, Sekip, Yogyakarta, 55281 Indonesia; 2 Dr. Sardjito General Hospital, Yogyakarta, Indonesia

**Keywords:** Monocyte-to-HDL ratio, Cardio ankle vascular index, Arterial stiffness, Inflammation biomarkers, Diabetes

## Abstract

**Background:**

Previous studies proposed that chronic inflammation in diabetes has a role in abnormal collagen production and elastin degradation, which promotes arterial stiffness. Monocyte-to-High Density Lipoprotein cholesterol ratio (MHR) is a simple measurement associated with inflammation and oxidative stress. However, little is known about the relationship of MHR with arterial stiffness. This study aimed to determine the association of MHR with arterial stiffness in patients with diabetes.

**Methods:**

A total of 81 patients with type 2 diabetes mellitus were enrolled in a cross-sectional study. Arterial stiffness factor in this study was Cardio Ankle Vascular Index (CAVI). We analyzed complete blood count and lipid profile in all participants, then performed statistical analysis to determine the relationship between MHR and CAVI. Receiver operating characteristic (ROC) analysis was used to estimate the cut-off values of MHR to predict CAVI ≥ 9.

**Results:**

Median of MHR in this study was 11.91 with the mean of CAVI was 8.13 ± 0.93. Spearman correlation analysis revealed a significant positive correlation between MHR and CAVI (ρ = 0.239, *p* = 0.031). Multivariate analysis showed the independent association of MHR to arterial stiffness (β = 0.361, 95% CI 0.023–0.093) and to CAVI ≥ 9 (OR 1.181, 95% CI 1.047–1.332). The cut-off values of MHR for predicting CAVI ≥ 9 were identified as ≥ 13 (OR 3.289, 95% CI 1.036–10.441).

**Conclusion:**

MHR is associated with CAVI in patients with diabetes, irrespective of various potential confounders.

## Background

Patients with diabetes mellitus (DM) are at higher risk of vascular complications. The dysfunction of endothelial and smooth muscle cells causes vascular homeostasis alteration, becoming a pro-inflammatory/thrombotic condition. Hyperglycemia, gather with other risk factors such as arterial hypertension and dyslipidemia, increases the risk of macro and microvascular diabetic complications [1, 2]. Type 2 DM, especially the uncontrolled condition, associated with a risk of arterial stiffness in the future life [3]. Diabetes mellitus associated with aortic stiffness was found comparable to over 6 years of nonsmoker without diabetes [4].

Arterial stiffness is the result of complex interactions of cellular and structural elements of vessel walls. Imbalance of vessel stability, especially through inflammation stimulation, causes an excess production of abnormal collagen and elastin diminution [5]. Arterial stiffness is found as an independent predictor of cardiovascular events [6]. The multicentre observational Rebound study showed that the increasing of arterial stiffness predict the risk of all-cause and cardiovascular mortality in type 2 diabetes [7]. Another study demonstrated the association of arterial stiffness with subclinical myocardial injury in patients with type 2 DM [8]. Therefore, the assessment of arterial stiffness in patients with type 2 DM is important to decide the best management to prevent future cardiovascular events.

Inflammation has a significant role in major arterial stiffness. Macrophages and monocytes produce pro-inflammatory and pro-oxidant cytokines in areas with inflammation. The increasing of monocyte count occurs in the worst cardiovascular diseases [9]. High Density Lipoprotein (HDL) has already been known as a particle that protects endothelial cells [10]. HDL stimulates Nitrite oxide (NO) release and increases the expression of eNOS (endothelial NO synthase). HDL suppresses the expression of adhesion molecules, such as vascular cell adhesion molecule 1, and inhibits the adhesion of white blood cells. HDL also has antithrombotic effect since it reduces the expression of tissue factor in endothelial cells exposed to cytokines and reduces platelet activation [11]. Monocyte to HDL ratio (MHR) is a new biomarker related to inflammation and oxidative stress, shown as the increasing of monocyte count in proportion to the reducing of HDL cholesterol level. Previous study showed that MHR positively correlates with diabetes mellitus and significantly associated to resistant hypertension in chronic kidney disease patients [12]. The increasing of MHR has also known as a biomarker of diabetic nephropathy [13]. However, the role of MHR in arterial stiffness of patients with diabetes is still not well-explored. The aim of this study is to observe the association of MHR with arterial stiffness in patients with diabetes.

## Methods

### Research design and subjects

The research design was an observational study with cross-sectional methods. We conducted a consecutive sampling to enroll patients who were diagnosed with DM. The study was conducted in the outpatient clinic of Endocrinology Clinic and Cardiology Clinic of Dr. Sardjito General Hospital, Yogyakarta, Indonesia. The research was performed from April to July 2020. The inclusion criteria of this study were patients who were diagnosed as DM type 2 and willing to enroll in this study. Patients with infection, haematology malignancy, autoimmune disease, on oral or intravenous corticosteroid treatment, chronic kidney disease (estimated Glomerular Filtration Rate (eGFR) < 60 mL/min/1.73 m^2^), chronic liver disease, peripheral artery disease (Ankle Brachial Index (ABI) < 0.9), pregnancy, with permanent pacemaker, and patients who were unable to be examined (unable to walk without device, with respiratory support, after limb amputation, and/or limb fracture) were excluded from the study. This study was approved by the Medical and Health Research Ethics Committee (MHREC) Faculty of Medicine, Public Health and Nursing, Universitas Gadjah Mada—Dr. Sardjito General Hospital, Indonesia.

### Clinical assessments

Medical history was collected by interviewing the patients and obtaining the medical record of each patient. Diabetes mellitus was diagnosed as fasting glucose plasm ≥ 126 mg/dL, or plasm glucose ≥ 200 mg/dL 2 h after oral glucose tolerance test with 75 mg glucose load, or random plasm glucose ≥ 200 mg/dL with the classical symptom, or HbA1c ≥ 6.5%. Uncontrolled DM was defined as HbA1c ≥ 7% from peripheral blood measurement. Smoking status, DM duration, and statin treatment duration were identified by anamnesis. Smoking status was classified as an active smoker, former smoker, and non-smoker [14]. DM duration was calculated from the first time patient diagnosed with DM. Body mass index (BMI) was calculated by body weight/(height^2^). Systolic blood pressure and pulse pressure were measured using the same device for all patients. Hypertension was defined as average blood pressure more than 140/90 (after three times measurements), or in the treatment of antihypertensive. Treatments were collected from the medical records. Statin treatment was classified as a low-intensity statin, moderate-intensity statin, high-intensity statin, and high-intensity statin added by ezetimibe[15]. DM complications were identified from the diagnosis of coronary artery disease, stroke, chronic heart failure, or diabetic retinopathy.

### CAVI and laboratory data collection

CAVI was measured by Vascular Screening System VaSera VS-1500 (Fukuda Denshi, Japan). The patient was in supine position at least 10 min before examination. Arm and ankle cuff were wrapped and microphone for phonocardiograph was seated on 2nd intercostal space of sternum. ABI and CAVI results were shown automatically in the VaSera monitor. ABI was calculated as the lowest ABI measured from right and left ankle. CAVI was measured as mean of right and left CAVI. The antecubital vein blood samples were taken after fasting for at least 8 h, and analyzed in the central hospital laboratory. Complete leucocyte count, HDL and LDL cholesterol level, fasting blood glucose, HbA1c and creatinine were measured. Monocyte-to-HDL ratio (MHR) was calculated manually by dividing monocyte count and HDL cholesterol level. Blood examinations were done in the same day as CAVI examination. The primary outcome of this study was the association between MHR and arterial stiffness, as measured by CAVI. The secondary endpoint was the optimal cut-off value of MHR to predict CAVI ≥ 9, as as the predictive cut-off for the future cardiovascular events in asymptomatic patients with type 2 diabetes [16].

### Statistical analysis

Statistical analysis was performed using SPSS IBM 23 version (International Business Machine Corp., Chicago) for Windows. The estimation of minimal sample size was 81 patients with the statistical power of 75%. The estimated sample size was calculated for the correlation of MHR with arterial stiffness. Normality test was analyzed by Kolmogorov Smirnov test, in which *p* > 0.05 was considered normal distribution data and shown as mean ± standard deviation (SD), while non-normal distribution data were shown as median with interquartile range (IQR). Categorical data were shown as number (percentage). Correlations between two numeric data were analyzed by Spearman correlation tests for non-normal distribution data. The difference of CAVI mean with each categorical variable was analyzed by independent t-tests. The difference of MHR distribution with each categorical variable was analyzed by Mann Whitney test. Univariate linear regression was assessed and multivariate linear regression was conducted for the variables that have significance p < 0.25 in univariate analysis, for the assessment of association of variables with arterial stiffness. Result of *p* < 0.05 in multivariate linear regression was considered as statistically significant. Logistic regression was performed in order to observe the association of MHR with CAVI ≥ 9. The receiver operating characteristic (ROC) curve was used to demonstrate the sensitivity, specificity, and cut-off value of MHR.

## Results

**Fig. 1:**
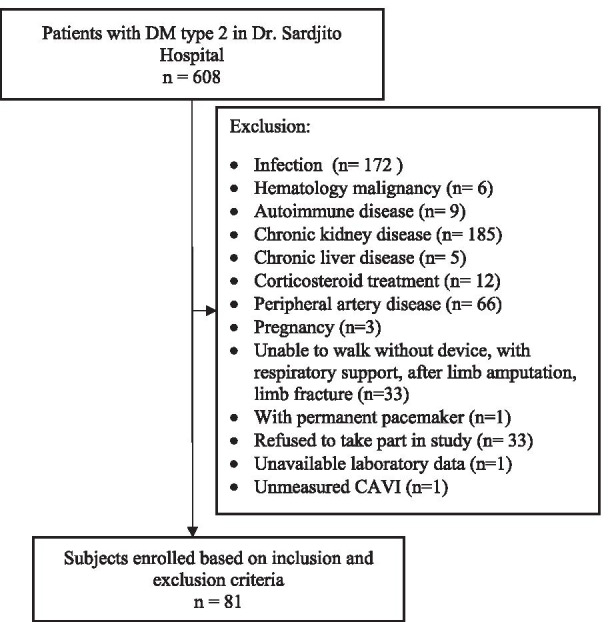
Flowchart of participant inclusion. CAVI, Cardio Ankle Vascular Index; ABI, Ankle Brachial Index; eGFR, estimated Glomerular Filtration Rate

From April to July 2020, 608 patients with diabetes presented at Dr. Sardjito General Hospital Yogyakarta, Indonesia. Five hundred and twenty seven patients were excluded based on the above mentioned criteria, and 81 subjects was enrolled in this study (Fig. 1).

**Fig. 2:**
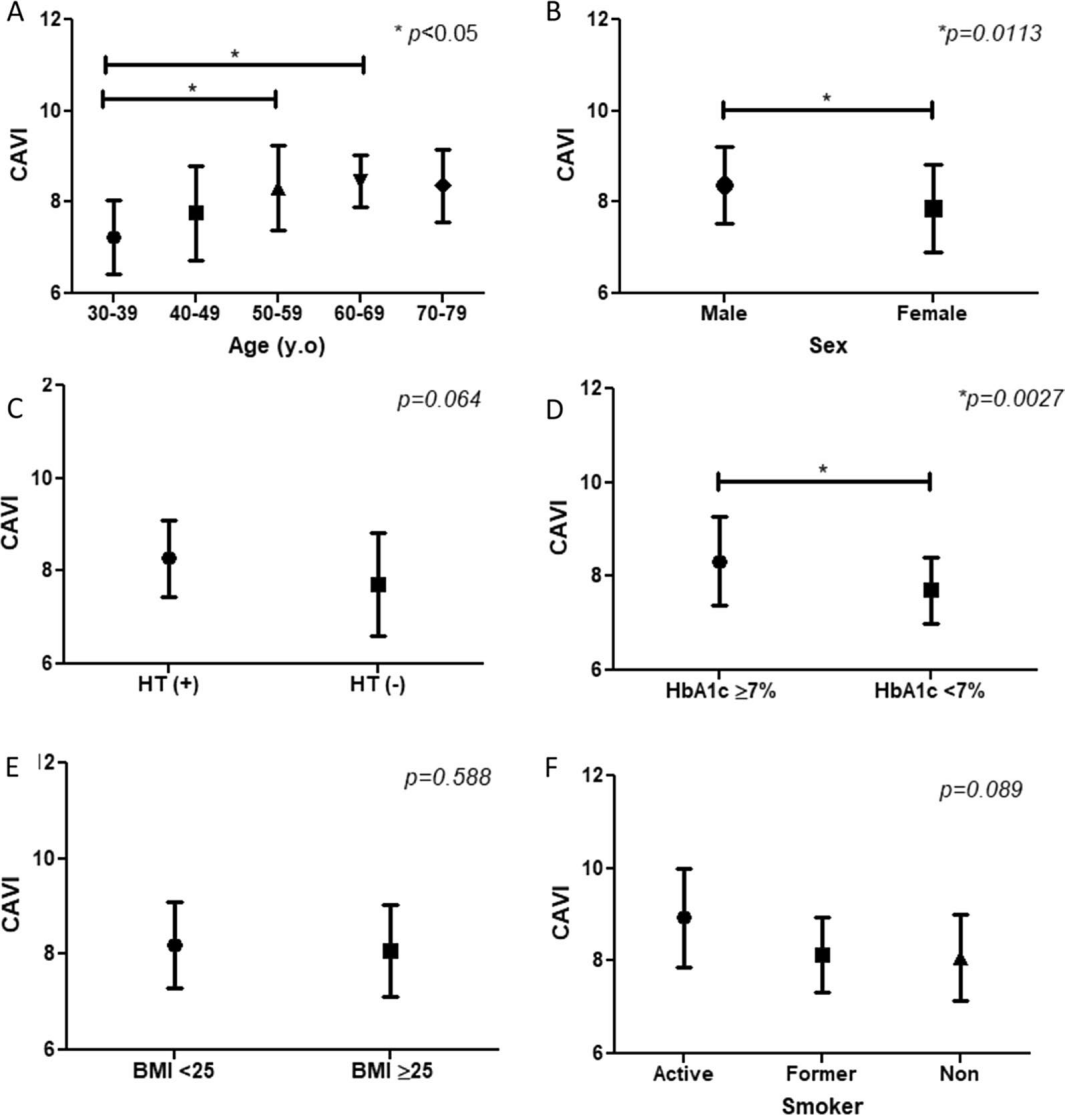
Comparison of mean CAVI according to the increasing of age (**A**), sex (**B**), hypertension status (**C**), uncontrolled diabetes (**D**), BMI (**E**), and smoker (**F**). CAVI, Cardio Ankle Vascular Index; BMI, Body Mass Index

**Fig. 3:**
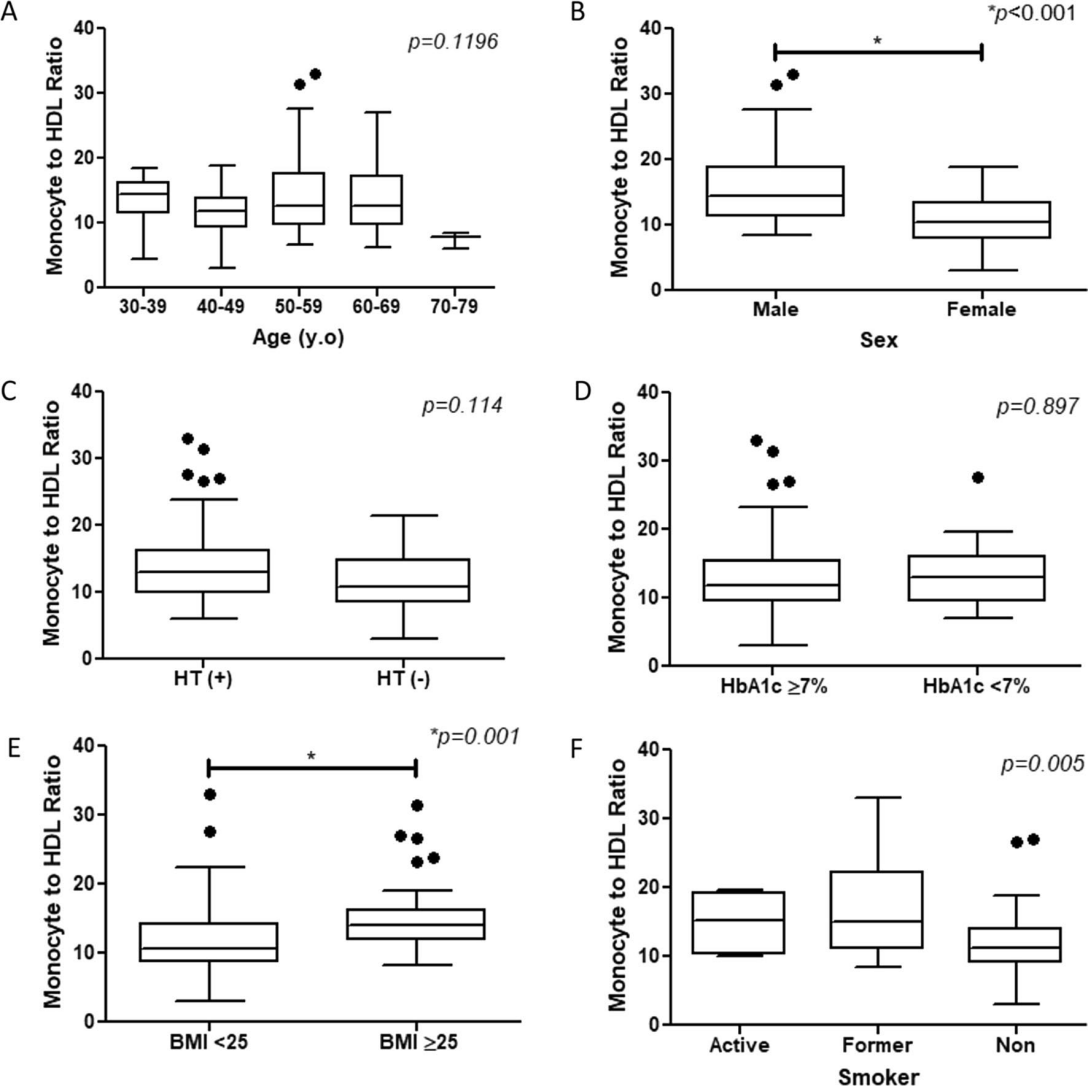
Comparison of monocyte-to-HDL ratio distribution according to the increasing of age (**A**), sex (**B**), hypertension status (**C**), uncontrolled diabetes (**D**), BMI (**E**), and smoker (**F**). CAVI, Cardio Ankle Vascular Index; BMI, Body Mass Index

This study consisted of patients with DM with the age range from 30 to 77 years old with median 55 years old. Among the subjects, 45 patients were male (55.6%). Baseline characteristics of this study are shown in Table 1. The mean of CAVI increased in age 30 to 69, was higher in male, uncontrolled diabetes and in active smoker (Fig. 2). Meanwhile, MHR distribution was higher in male and high BMI patients (Fig. 3).

**Table 1:** Baseline characteristics of the subject participants

Variable (n = 83)	Value
Age, y.o	55 (49–60)
*Sex*	
Male, n (%)	45 (55.6)
Female, n (%)	36 (44.4)
Smoking, n (%)	
Active smoker, n (%)	6 (7.4)
Former smoker, n (%)	24 (29.6)
Never smoker, n (%)	51 (63)
DM duration, years	6 (3–11)
History of hypertension, n (%)	52 (64.2)
History of dyslipidemia, n (%)	48 (59.3)
DM complication:	44 (54.3)
Coronary artery disease, n (%)	28 (34.6)
Chronic heart failure, n (%)	7 (8.6)
Stroke, n (%)	9 (11.1)
Diabetic retinopathy, n (%)	13 (16)
Body mass index, kg/m^2^	24.78 ± 3.79
Systolic blood pressure, mmHg	141.12 ± 20.49
Pulse pressure, mmHg	58.36 ± 15.36
*Treatment parameter*	
Statin, n (%)	48 (59.3)
Low intensity statin, n (%)	17 (21)
Moderate intensity statin, n (%)	28 (34.6)
High intensity statin, n (%)	3 (3.7)
High intensity statin + ezetimibe, n (%)	0
Statin duration, years	0.083 (0–1.5)
ACE inhibitor/ARB, n (%)	46 (56.8)
Vitamin, n (%)	40 (49.4)
Antidiabetic drugs:	79 (97.5)
Oral anti diabetic drugs, n (%)	35 (43.2)
Insulin, n (%)	27 (33.3)
Combination of oral and insulin, n (%)	17 (21)
Nitrate, n (%)	11 (13.6)
NSAID, n (%)	32 (38.3)
*Artery examination parameter*	
ABI	1.05 ± 0.076
CAVI	8.14 ± 0.93
*Laboratory parameter*	
Leucocyte count, × 10^3^/μL	8.06 ± 2.05
Monocyte count, × 10^3^/ μL	0.53 (0.46–0.64)
Fasting blood glucose, mg/dL	151 (113–210)
HDL cholesterol, mg/dL	43 (38–50)
LDL cholesterol, mg/dL	133.75 ± 41.68
LDL ≥ 160 mg/dL, n (%)	23 (28.4)
HbA1c, %	8.56 ± 1.98
HbA1c ≥ 7%, n (%)	58 (71.6)
Monocyte to HDL ratio (MHR)	12.56 (9.62–15.53)
Creatinine, mg/dL	0.89 ± 0.2
Estimated GFR, mL/min/1.73 m^2^	84.35 (71.51–101.09)

Data were shown as mean ± SD for normal distribution data, median (interquartile range) for non-normal distribution data and number (percentage) for categorical data

ABI: Ankle brachial index, ACE: angiotensin converting enzyme, ARB: angiotensin receptor blocker, CAVI: cardio ankle vascular index, DM: diabetes mellitus, GFR: glomerular filtration rate, HbA1c: hemoglobin a1c, HDL: high density lipoprotein, LDL: low density lipoprotein, NSAID: non steroid anti-inflammatory drugs, SD: standard deviation

**Fig. 4:**
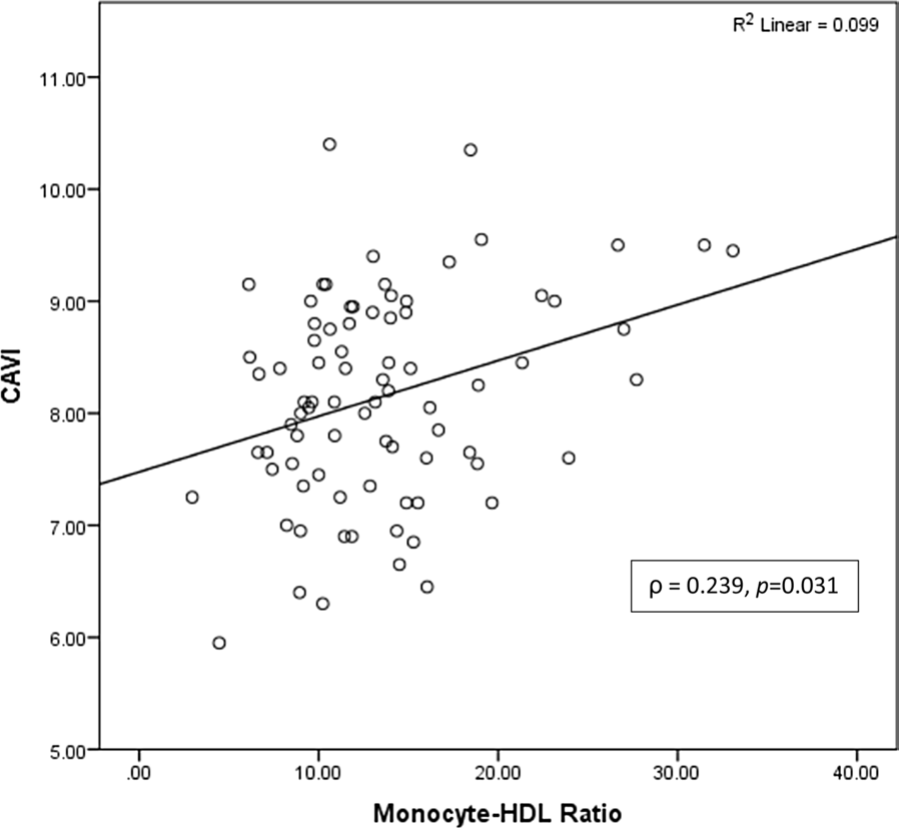
Correlation of monocyte-to-HDL ratio with cardio ankle vascular index. HDL, High Density Lipoprotein

Correlations of MHR with CAVI analysed by Spearman analysis were positive and significant correlations (ρ = 0.239, *p* = 0.031) (Fig. 4). In addition, CAVI negatively correlated with the HDL cholesterol levels (ρ = −0.284, *p* = 0.01), but not with the monocyte count (ρ = 0.156, *p* = 0.165). Univariate linear regression showed that age, body mass index, HbA1c, MHR, sex, smoking, hypertension, history of coronary artery disease, oral antidiabetic drugs and insulin had association with CAVI with *p* value < 0.25. Multivariate linear regression analysis revealed that all the variable values were associated with CAVI with R^2^ = 0.422, *p* = 0.000. Partially, age, body mass index, HbA1c, MHR and smoking had an independent association with CAVI (*p* < 0.05) (Table 2). The results of this analysis revealed that there is an independent association of MHR with arterial stiffness, as measured by CAVI.

**Table 2:** Univariate and multivariate linear regression to predict arterial stiffness

Variables	Univariate linear regression			Multivariate linear regression		
	Beta	95% CI	*p* value	Beta	95% CI	*p* value
Age	0.365	0.017 to 0.061	0.001	0.28	0.008 to 0.051	0.008*
DM duration	0.098	− 0.017 to 0.043	0.384			
Monocyte-to-HDL ratio	0.315	0.016 to 0.083	0.004	0.361	0.023 to 0.093	0.001*
BMI	− 0.183	− 0.097 to 0.009	0.102	− 0.281	− 0.119 to (− 0.017)	0.01*
HbA1c	0.144	− 0.037 to 0.172	0.203	0.24	0.023 to 0.203	0.015*
LDL	− 0.078	− 0.006 to 0.003	0.491			
Total cholesterol	0.199	− 0.004 to 0.01	0.340			
Sex	0.285	0.129 to 0.924	0.01	0.078	− 0.251 to 0.540	0.468
Smoking	0.244	0.093 to 1.618	0.028	0.197	0.027 to 1.357	0.042*
Hypertension	0.248	0.070 to 1.029	0.025	0.143	− 0.131 to 0.765	0.163
Coronary artery disease	0.143	− 0.152 to 0.705	0.203	− 0.042	− 0.489 to 0.325	0.688
Statin	− 0.078	− 0.564 to 0.271	0.486			
Vitamin	0.028	− 0.361 to 0.462	0.807			
NSAID	0.101	− 0.230 to 0.613	0.368			
ACE inhibitor/ARB	0.012	− 0.394 to 0.437	0.918			
Oral antidiabetic drugs	0.142	− 0.152 to 0.698	0.205	0.131	− 0.178 to 0.683	0.246
Insulin	− 0.139	− 0.667 to 0.152	0.214	− 0.203	− 0.803 to 0.05	0.083

*p < 0.05 was considered statistically significant

**Fig. 5:**
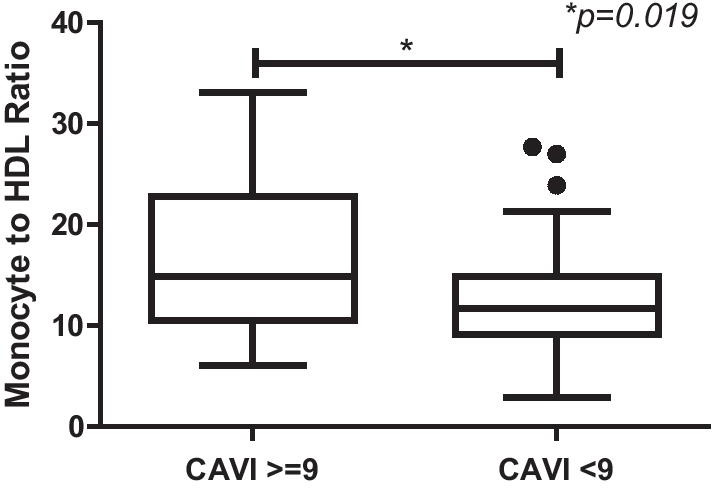
Distribution of monocyte-to-HDL ratio in CAVI ≥ 9 and CAVI < 9. HDL, High Density Lipoprotein; CAVI, Cardio Ankle Vascular Index

**Fig. 6:**
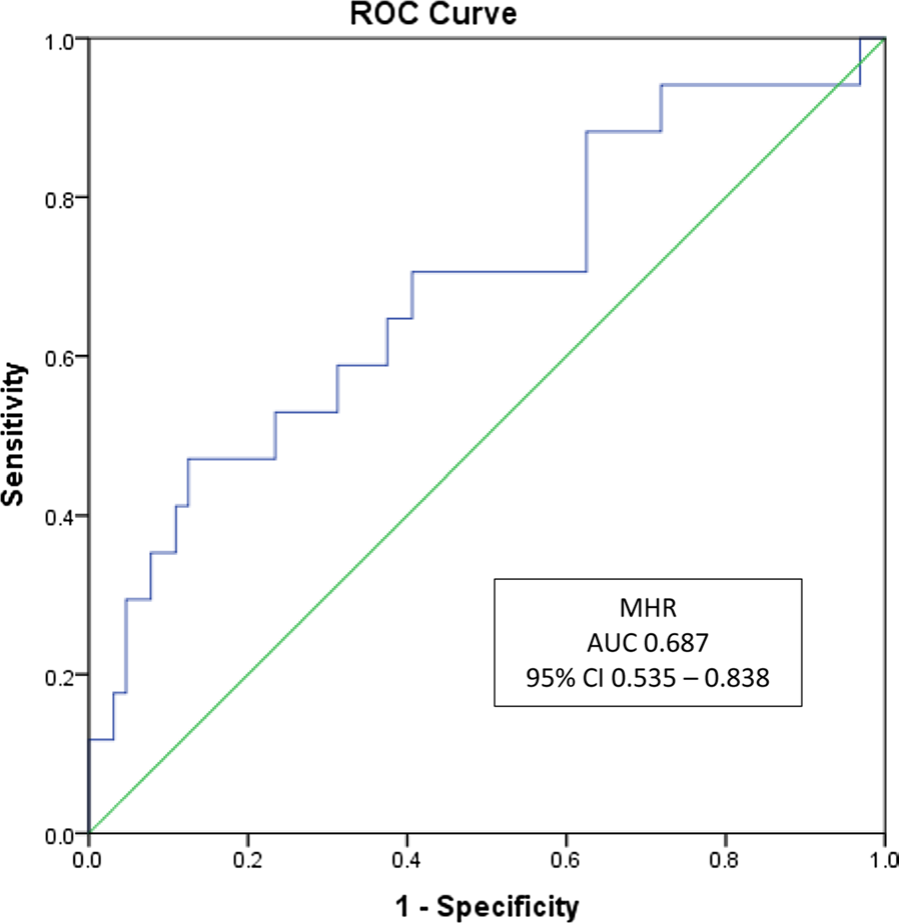
ROC curve analysis and AUC of MHR for prediction of CAVI ≥ 9. AUC, Area under curve; HDL, High Density Lipoprotein; CAVI, Cardio Ankle Vascular Index; MHR, Monocyte-to-HDL Ratio

There was a significant difference between MHR distribution in CAVI ≥ 9 and CAVI < 9 (*p* = 0.019) (Fig. 5). From multivariate logistic regression, MHR and HbA1c were independently associated with CAVI ≥ 9 (Table 3). ROC curve analysis showed that the AUC of MHR was 0.687 (95% CI 0.535–0.838) (Fig. 6). Monocyte-to-HDL ratio of ≥ 13 (OR 3.289, 95% CI 1036–10.441) had the best prediction cut-off value for CAVI ≥ 9 with sensitivity of 70.6% and specificity of 59.4%.

**Table 3:** Univariate and multivariate logistic regression predict CAVI ≥ 9

Variables	Univariate logistic regression			Multivariate logistic regression		
	OR	95% CI	*p* value	OR	95% CI	*p* value
Age	1.063	0.991–1.140	0.087	1.074	0.983–1.172	0.112
DM duration	1.003	0.928–1.084	0.944			
Monocyte-to-HDL ratio	1.138	1.037–1.248	0.006	1.181	1.047–1.332	0.007*
BMI	0.973	0.843–1.123	0.705			
HbA1c	1.292	0.981–1.702	0.069	1.515	1.044–2.199	0.029*
LDL	1.003	0.991–1.016	0.589			
Total cholesterol	1.010	0.992 –1.028	0.296			
Sex	0.444	0.140–1.405	0.167	0.855	0.189–3.873	0.855
Smoking	4.357	0.794–23.909	0.09	4.717	0.657–33.852	0.123
Hypertension	1.429	0.361–5.648	0.611			
Coronary artery disease	1.041	0.340–3.193	0.944			
Statin	0.977	0.330–2.899	0.967			
Vitamin	1.198	0.410–3.495	0.741			
NSAID	1.167	0.392–3.471	0.782			
ACE inhibitor/ARB	0.608	0.208–1.782	0.365			
Oral antidiabetic drugs	1.44	0.452–4.591	0.538			
Insulin	0.691	0.236–2.021	0.5			

*p < 0.05 was considered statistically significant

## Discussion

In this pilot study, we found that there was a positive correlation of MHR with arterial stiffness, which was measured by CAVI in patients with diabetes. Furthermore, MHR may be used as a diagnostic tool for the prediction of CAVI ≥ 9. However, the power of this study is 75%, which is considered underpowered. Therefore, further study with the larger sample size is needed.

There are only limited number of studies about arterial stiffness in Indonesia and this is the first study to observe the association of MHR with arterial stiffness, in which arterial stiffness is measured as CAVI using a VaSera machine. Arterial stiffness measurement by CAVI has some advantages such as it is independent of blood pressure at the time of measurement, has high reproducibility and sensitivity, and uses a user-friendly device with a simplified procedure [17]. CAVI is also an accurate predictor of cardiovascular events [18], and macrovascular complications such as peripheral artery disease in patients with diabetes who undergo dialysis [19]. CAVI that was used by this study is a combination of PWV and β index, in which β index assessed the stiffness only in one artery segment, but CAVI reflects the arterial stiffness of all arterial tree [20]. In the other study, there was a positive significant correlation between CAVI and β index [21].

The association of MHR with CAVI in this study supports the previous finding that showed the positive and statistically significant correlation of MHR with arterial stiffness (β index) in patients with untreated hypertension and negative correlation of MHR with aortic distensibility [22]. Other significant finding in this study was the different distribution of MHR in CAVI ≥ 9 and CAVI < 9. CAVI ≥ 9 consist of higher number of MHR than CAVI < 9. This result direct the chance that MHR associate with arterial stiffness, as measured by CAVI.

The independent relationship between MHR and CAVI showed the role of inflammation in the arterial stiffness process, as shown by monocytes’ pro-inflammatory function and HDL anti-inflammatory properties. Interconnection between inflammatory pathways and various large artery stiffening mechanisms (degradation of elastin, calcification of medial, endothelial dysfunction) is mediated by increasing inflammatory cells. Inflammatory cascades lead to endothelial dysfunction promote vascular smooth muscle cells osteogenic differentiation and calcification [23]. Monocytes are the important elements of the inflammatory process. In a healthy community, monocyte count is a predictor of subclinical carotid atherosclerosis [24]. In diabetes, there would be an increase in pro-atherogenic monocyte activity such as IL-6 and IL-1β production [25]. Another study revealed that there was an activation state of peripheral blood monocytes in diabetes with the increasing of CD36 and MCP-1 gene expressions [26]. Furthermore, in patients with diabetes, there is a strong polarization of monocytes caused by pro-inflammatory stimuli that promote the circulating monocytes into the activated M1 and M2 phenotypes [27]. Hyperglycaemia in diabetes will induce abnormal collagen production and elastin reduction that allows the formation of arterial stiffness [28]. Chronic inflammation also causes the decreasing of the number and function of HDL which will diminish its anti-inflammatory properties [29].

Monocyte-to-HDL ratio is a systemic inflammatory marker of various cardiovascular disorders. The MHR would be much higher in patients with type 2 diabetes [30] and metabolic syndrome [31, 32]. The higher MHR in patients with diabetes was supported by the increase of monocyte [33] and the decrease of HDL levels compared to non-diabetic patients [34, 35]. Arterial stiffness and MHR may be linked by a subclinical inflammation in the vessel wall. The increasing of white blood cell counts, including the monocyte count, had a dose-dependent association with brachial-ankle pulse wave velocity tertiles in patients with type 2 diabetes mellitus in China [36]. On the other side, HDL had a negative correlation with CAVI in hypertension patients [37] and with abdominal aortic stiffness assessed by β stiffness index in subjects with varying insulin sensitivity [38].

Inflammation and lipid abnormality are main factors in the development of atherosclerosis, have role in healing response to vascular injury and allow the initiation and growth of atherosclerotic plaque [39]. Increasing of arterial stiffness is an indicator of atherosclerosis process in the vessel wall, and this arterial stiffness has a high predictive value in the future cardiovascular events [40]. Previous study showed an association of baPWV and augmentation index with inflammatory markers, such as CRP, tumor necrosis factor-alpha (TNF-α) and Interleukin-6 (IL-6) in hypertension patients [41] and CRP in healthy individuals [42].

Other earlier studies observed the correlation of MHR with hs-CRP and found it was associated with slow coronary flow events of stable angina pectoris with normal coronary arteries by angiography [43]. Other study observed that MHR positively correlates to CRP, Gensini score and SYNTAX score in patients with acute coronary syndrome. Furthermore, MHR in third tertile group is an independent predictor to cardiovascular events during hospitalization and long term follow-up [44, 45]. Meanwhile in patients with diabetes, MHR was also correlated with carotid intimal-media thickness [30] and strongly associated with coronary artery disease in type 2 DM [46].

Some limitations of this study included the small number of the samples which limited the significance of the findings. The ROC analysis to define the cut-off was still based on the cross-sectional study for piloting the study. Further study with a cohort prospective design is needed. Besides, in this study there were no other inflammation markers examined such as CRP, IL-6 and TNF-α which can be used to confirm the role of MHR in arterial stiffness.

## Conclusion

Our findings indicate that MHR is associated with arterial stiffness in diabetic patients, irrespective of various potential confounders. A cut-off MHR ≥ 13 may predicts the CAVI ≥ 9. Future study is needed to observe the benefit of MHR measurement in the prediction of arterial stiffness in patients with diabetes.

## Data Availability

The data of this study may be available on reasonable request to the corresponding author.

## Abbreviations

HDL: High density lipoprotein
MHR: Monocyte-to-HDL ratio
CAVI: Cardio ankle vascular index
DM: Diabetes melitus
NO: Nitrite oxide
eNOS: endothelial NO synthase
LDL: Low density lipoprotein
ABI: Ankle brachial index
eGFR: estimated Glomerular filtration rate
SD: Standard deviation
IQR: Interquartile range
AUC: Area under curve
PWV: Pulse wave velocity
baPWV: Brachial ankle pulse wave velocity
IL-1β: Interleukin-1 beta
IL-6: Interleukin-6
MCP-1: Monocyte chemoattractant protein-1
CRP: C-reactive protein
TNF-α: Tumor necrosis factor-alpha
